# Leukemia Inhibitory Factor Protects Axons in Experimental Autoimmune Encephalomyelitis via an Oligodendrocyte-Independent Mechanism

**DOI:** 10.1371/journal.pone.0047379

**Published:** 2012-10-15

**Authors:** Melissa M. Gresle, Estella Alexandrou, Qizhu Wu, Gary Egan, Vilija Jokubaitis, Margaret Ayers, Anna Jonas, William Doherty, Anna Friedhuber, Gerry Shaw, Michael Sendtner, Ben Emery, Trevor Kilpatrick, Helmut Butzkueven

**Affiliations:** 1 Department of Medicine; The Melbourne Brain Centre at the Royal Melbourne Hospital, The University of Melbourne, Parkville, Victoria, Australia; 2 Florey Neuroscience Institutes, Parkville, Victoria, Australia; 3 Department of Anatomy, The University of Melbourne, Parkville, Victoria, Australia; 4 Monash Biomedical Imaging & School of Psychology and Psychiatry, Monash University, Clayton, Australia; 5 Department of Pathology, The University of Melbourne, Parkville, Victoria, Australia; 6 McKnight Brain Institute, University of Florida College of Medicine, Gainesville, Florida, United States of America; 7 Institute for Clinical Neurobiology, University of Wurzburg, Wurzburg, Germany; Schepens Eye Research Institute, Harvard Medical School, United States of America

## Abstract

Leukemia inhibitory factor (LIF) and Ciliary Neurotrophic factor (CNTF) are members of the interleukin-6 family of cytokines, defined by use of the gp130 molecule as an obligate receptor. In the murine experimental autoimmune encephalomyelitis (EAE) model, antagonism of LIF and genetic deletion of CNTF worsen disease. The potential mechanism of action of these cytokines in EAE is complex, as gp130 is expressed by all neural cells, and could involve immuno-modulation, reduction of oligodendrocyte injury, neuronal protection, or a combination of these actions. In this study we aim to investigate whether the beneficial effects of CNTF/LIF signalling in EAE are associated with axonal protection; and whether this requires signalling through oligodendrocytes. We induced MOG_35–55_ EAE in CNTF, LIF and double knockout mice. On a CNTF null background, LIF knockout was associated with increased EAE severity (EAE grade 2.1±0.14 vs 2.6±0.19; P<0.05). These mice also showed increased axonal damage relative to LIF heterozygous mice, as indicated by decreased optic nerve parallel diffusivity on MRI (1540±207 µm^2^−/s vs 1310±175 µm^2^−/s; P<0.05), and optic nerve (−12.5%) and spinal cord (−16%) axon densities; and increased serum neurofilament-H levels (2.5 fold increase). No differences in inflammatory cell numbers or peripheral auto-immune T-cell priming were evident. Oligodendrocyte-targeted gp130 knockout mice showed that disruption of CNTF/LIF signalling in these cells has no effect on acute EAE severity. These studies demonstrate that endogenous CNTF and LIF act centrally to protect axons from acute inflammatory destruction via an oligodendrocyte-independent mechanism.

## Introduction

In Multiple Sclerosis (MS), progressive neurological decline is, at least partially, associated with the cumulative and irreversible loss of axons in the CNS. Current disease modifying therapies for MS, which are largely targeted towards the modulation of peripheral immune cell activity, are incompletely effective against disability progression in this disease. This likely reflects the complex mechanisms of axonal pathology in MS that not only comprises peripheral immune-cell mediated injury [1], [2], [3], but also damaging events mediated by resident CNS glia [4], [5], re-myelination failure [6], and maladaptive responses in demyelinated axons [7], [8]. Hence, combination therapies are likely to be required to minimize axonal damage and the progression of disability in this disease.

In humans, microarray analyses of non-lesioned motor cortex tissue have shown that components of the Ciliary neurotrophic factor (CNTF)/Leukemia inhibitory factor (LIF) signalling pathway are more highly expressed in MS patients than controls, potentially representing an endogenous protective response to limit neural injury in neuro-inflammatory diseases [9]. Both LIF and CNTF are members of the interleukin-6 family of cytokines, which signal through the common receptor subunit glycoprotein 130 (gp130) and activate several downstream signalling cascades including Janus kinase/signal transducer and activator of transcription, nuclear factor kappa-light-chain-enhancer of activated B cells, mitogen activated protein kinase and AKT/PI3-kinase. In neural cells, Both cytokines have been shown to promote survival of neurons and oligodendrocytes in vitro [10], [11], [12], [13], [14], [15], improve neuronal survival following axotomy in vivo [14], [16], [17], and modulate inflammatory cell activity [18], [19], [20]. Considerable functional overlap between CNTF and LIF has been described, which is explained by their sharing of the signal transducing receptor subunits gp130 and LIF receptor beta [21].

Importantly, the therapeutic administration of both CNTF and LIF has been shown to improve neurological outcome in the murine experimental autoimmune encephalomyelitis (EAE). In the SJL/J EAE model, treatment with recombinant murine LIF was shown to reduce oligodendrocyte apoptosis and demyelination, with no obvious influence on inflammatory cell infiltrates in these mice [22]. In contrast, daily treatment with recombinant CNTF was shown to reduce the formation or persistence of inflammatory cell infiltrates in the CNS, which was associated with reduced damage to neurons, axons and oligodendrocytes [23]. Interestingly, although the latter study provides some evidence that endogenous CNTF can reduce neuronal damage during EAE, conditional gp130 deletion experiments in neurons suggest that neuronal gp130 signalling does not influence EAE outcome, whereas astrocytic gp130 signalling does [24].

At present, it is uncertain if endogenous LIF and CNTF act through oligodendrocytes in conferring EAE protection, if their activities are redundant in EAE, and if their ultimate protective effect is achieved through reduction in axonal injury. We therefore assessed if LIF/CNTF double knockout mice exhibit more severe EAE disease; if endogenous gp130 signalling acts via oligodendrocytes in EAE; and If LIF/CNTF signalling reduces inflammatory axonal injury and loss in EAE.

## Materials and Methods

### Ethics Statement

All experiments were performed in accordance with guidelines issued by the National Health and Medical Research Council of Australia, and with approval from the Florey Neuroscience Institutes (Howard Florey Institute) animal ethics committee.

### Experimental Mouse Colonies

The CNTF/LIF gene knockout (KO) (Generated by Michael Sendtner [13]) mice were backcrossed on to a C57B/6 background for greater than 10 generations, and maintained at the Florey Neuroscience Institutes, Parkville, Australia. Given the well-known cage-to-cage and colony-to-colony variability of EAE, we conducted all EAE experiments on littermates housed together from birth and sex-segregated at weaning. As LIF KO mice are not born in Mendelian ratios (approximately 1 KO for every 5 heterozygotes (HET)) and LIF KO females are infertile, it was therefore imperative to generate four separate breeding colonies for these studies in order to obtain sufficient mice for experimental cohorts: Colony 1 CNTF wild type (WT)/LIF HET males bred with CNTF WT/LIF WT females, Colony 2 CNTF WT/LIF KO males bred with CNTF WT/LIF HET females, Colony 3 CNTF KO/LIF HET males bred with CNTF KO/LIF WT females, and Colony 4 CNTF KO/LIF KO males bred with CNTF KO/LIF HET females. Importantly, we avoided comparisons between the experimental colonies, as these were maintained as separate lines.

Myelin basic protein (MBP)-cre mice (generated by Ben Emery [25], based on the 2kb MBP promotor from [26]) were crossed with floxed gp130 mice (generated by Werner Mueller [27]) to generate MBP-cre^+^ gp130^fl/fl^ mice and MBP-cre^−^ gp130^fl/fl^ mice for experimental cohorts. Both lines were backcrossed onto a C57B6 background for greater than 10 generations, and breeding colonies were maintained at the Florey Neuroscience Institutes (Parkville, Australia). The deletion efficiency of the Gp130 transcript from oligodendrocyte lineage cells derived from these mice was greater than 90% (see Data S1 for methods and Data S2 for qPCR analysis).

### Experimental Autoimmune Encephalomyelitis (EAE)

EAE was induced in male and female mice at 8–13 weeks of age (colony 1: n = 20 CNTF WT/LIF WT vs n = 9 CNTF WT/LIF HET; colony 2: n = 21 CNTF WT/LIF KO vs n = 30 CNTF WT/LIF HET; colony 3: n = 17 CNTF KO/LIF HET vs n = 19 CNTF KO/LIF WT; colony 4: n = 18 CNTF KO/LIF KO vs n = 30 CNTF KO/LIF HET; MBP-cre +ve gp130^fl/fl^ n = 33 vs MBP-cre -ve gp130^fl/fl^ n = 47) The age difference between mice in any given experiment was generally less than 3 weeks. Mice were immunized with MOG 35–55 (MEVGWYRSPFSRVVHLYRNGK) peptide (HD biosciences, Beijing, CN) as previously described [28], and assessments of EAE severity were performed daily according to a standardized paraplegia scale [22], [29], [30] consisting of 9 points:0 = no disease; 1 = tail weakness; 1.5 = tail weakness and slight gait abnormality; 2 = tail paralysis; 2.25 = tail paralysis and slight gait abnormality; 2.5 = tail paralysis and hindlimb paraparesis; 2.75 = paralysis tail of one hindlimb; 3 = complete hindlimb paralysis; 4 = quadraparesis or moribund (mice are euthanized at this stage). All mice referred to as healthy, are naive to immunization.

### Magnetic Resonance Imaging (MRI)

**Figure 1:**
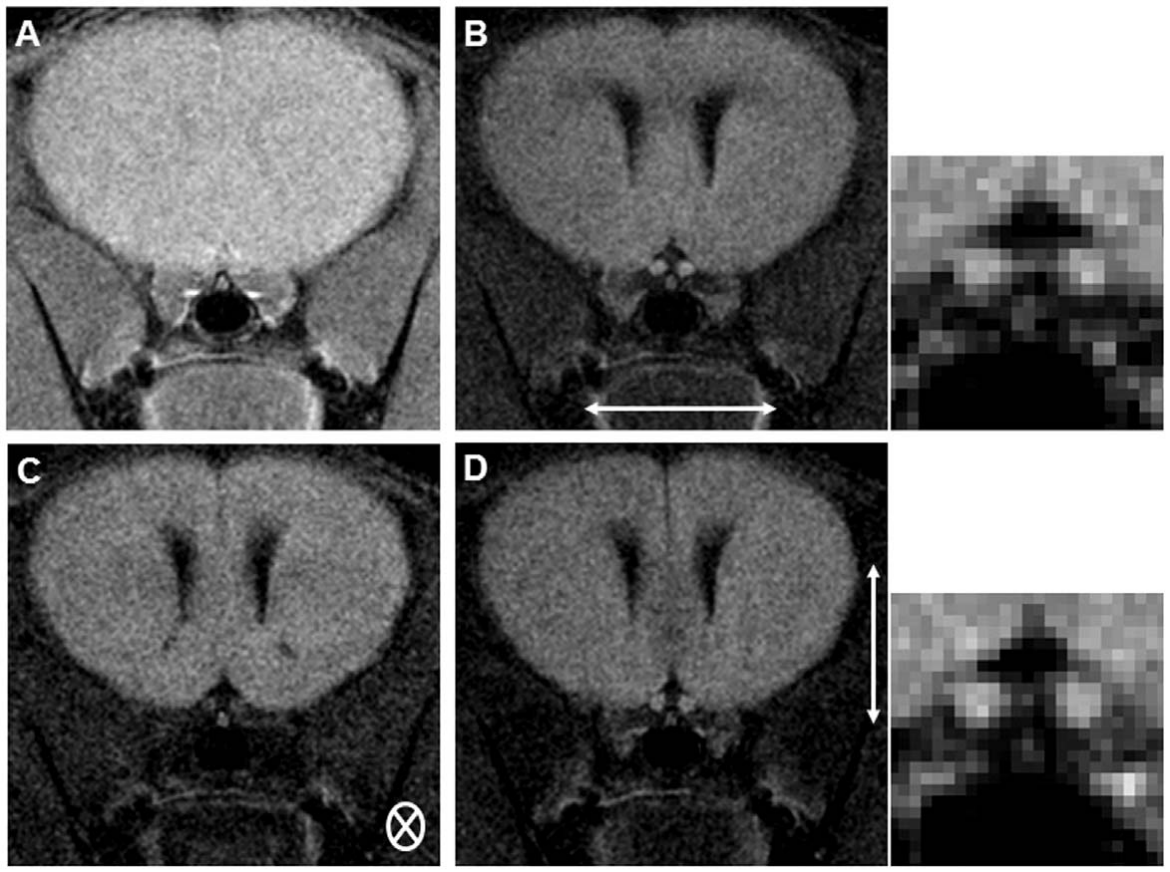
Coronal optic nerve diffusion imaging, pre-chiasmal intracranial optic nerve segment. The T2 weighted localiser scan is shown in (A). Diffusion weighted images perpendicular to the optic nerve are shown in (B and D), and images parallel to the optic nerve are shown in (C). B’ and D’ show enlargements of the optic nerve images. Diffusion direction is indicated by small white arrows.

The MRI scans were performed on n = 8 CNTF KO/LIF KO EAE induced mice, n = 10 CNTF KO/LIF HET EAE induced mice, and n = 8 healthy CNTF KO/LIF KO and CNTF KO/LIF HET mice per group, as previously described [31], using a Bruker Biospec 4.7T MRI scanner equipped with a 6-cm inner diameter actively shielded gradient coil capable of producing pulse gradients of up to 95 Gauss/cm. Scanning was performed between EAE day 18 and 21. For scanning, mice were anesthetized with 3% isoflurane in medical oxygen and placed in a MR-compatible head holder with a nose cone positioned to maintain anaesthesia with 0.5%–1% isoflurane. A 3 cm inner diameter column coil was used as a transmitter and receiver of the MR signal. To assess axonal injury within the optic nerves, diffusion-weighted imaging (DWI) was performed according to methods previously validated by Wu *et al* [31]. Importantly in their study it was shown that the apparent diffusion coefficient (ADC) parallel measure was strongly associated with histological evidence of axonal loss in the optic nerve (Pearson R = 0.75, P<0.01). In brief, both nerves were scanned in the coronal plane (Fig. 1A) with the diffusion encoding gradient directions set perpendicular (Fig. 1B and D) and parallel (Fig. 1C) to the pre-chiasmal portion of the optic nerve. Other parameters used in the DWI sequence: TR/TE = 800/25 ms, Δ = 10 ms, δ = 5 ms, 12 NEX, slice thickness = 1.0 mm, FOV = 1.5×1.5 cm^2^, matrix size 192×192 and b values of 0 and 700 s/mm^2^.

Diffusion weighted images were analysed on an off-line PC workstation using the image processing software MIStar (Apollo Medical Imaging Technology, Melbourne, AU). To minimize noise effects due to low signal-to-noise ratio associated with the small imaging pixel size, raw images were spatially filtered with a 3×3 Gaussian kernel (σ = 0.5) and then Apparent Diffusion Coefficient (ADC) maps were generated by fitting the image intensity decay on a pixel by pixel basis. Region of Interest (ROI) analyses of optic nerves, containing 10 pixels, were then performed to calculate the directional ADC values of the optic nerves. Each optic nerve was treated as a different sample.

### Neurofilament-Heavy Enzyme-linked Immunosorbant Assay for Axonal Damage

In order to further quantitate CNS axonal injury, we also assessed serum levels of the axonal cytoskeletal protein phosphorylated neurofilament-H (pNF-H), measured by enzyme-linked immunosorbant assay (ELISA). Importantly, in EAE mice, serum pNF-H levels have previously been shown to be strongly associated with spinal cord axon numbers and disease severity grades [28]. For collection of serum, immediately following MRI scanning at EAE day 18–21, mice were anesthetized with 100 mg/kg sodium pentobarbital and blood samples were obtained from the right atrium of mice prior to intracardiac perfusion (n = 11 CNTF KO/LIF KO and n = 12 CNTF KO/LIF HET EAE induced mice). The samples were centrifuged at 5000 rpm for 10 min at 4°C, and serum was collected for analysis. The serum pNF-H ELISA was then conducted according to methods previously described [28].

### Methylene Blue Axonal Histology

For quantification of axon numbers, we assessed methylene blue stained optic nerve and spinal cord cross-sections. Immediately following MRI scanning at EAE day 18–21, the mice were anesthetized with sodium pentobarbital (100 mg/kg, i.p) and intracardially perfused with 20 ml 0.1 M PBS (pH 7.4), followed by 20 ml of Karnovski’s buffer (0.1 M sodium cacodylate solution, pH 7.3, containing 2.5% gluteraldehyde and 4% paraformaldehyde). Both optic nerves and lumbar spinal cord tissue were collected for histology under a dissecting microscope. The tissue was then processed for Spurr’s resin embedding, cross-sectioned at 0.5 µm thickness using an ultramicrotome, and stained with methylene-blue solution, as previously described [28].

#### Optic nerve analyses

For axonal counts in optic nerve sections (n = 9 CNTF KO/LIF KO and n = 11 CNTF KO/LIF HET EAE induced mice, and n = 5 or 6 healthy mice per group), the centre of each methylene-blue stained nerve cross-section was photographed at 100 times magnification, corresponding to an area of 3480 µm^2^, and the whole optic nerve at 20 times magnification on a Zeiss Axioplan 2 Imaging system (Axiovision Software Release 4.4; Carl Zeiss Pty Ltd, Oberkochen, DE). Images were saved in TIFF format and imported into ImageJ (U.S. National Institutes of Health Freeware http://rsb.info.nih.gov/ij/) to be cropped. For nerves with substantial peripheral immune cell infiltration (lesion size greater than 3480 µm^2^), an additional area located adjacent to the primary region of interest was also captured and analysed.

For axon counts, the axons in each section were selected (indicated by filled axoplasma) and counted using the counts/size function in ImagePro (version 6.0.0.26; Media Cybernetics Inc., Bethesda, MD) by setting ranges for area (0.1–12), radius ratio (1–10) and roundness (0–3) for optic nerve images. Regions that did not include axons (inflammatory cells and lesions) were deselected manually (using ImagePro). Whole nerve axon counts for nerves that showed no substantial inflammatory cell infiltrates were calculated using the ratio of total nerve area/section area (Region of interest (ROI) = 3480 µm^2^) as a scaling factor. If inflammation was present, an estimate of axon counts was calculated by averaging the ratio of total central area/section area (ROI = 3480 µm^2^), and the ratio of total area of peripheral-infiltrated section/section area (ROI = 3480 µm^2^). This method was validated against manual counting and the average difference between the two methods was 152.8±95.1 axons/section (Data S3 A).

#### Spinal cord analyses

For analyses of axonal numbers in the lumbar spinal cord (n = 10 CNTF KO/LIF KO and n = 12 CNTF KO/LIF HET EAE induced mice, and n = 3 healthy mice per group), two images of the dorsal column, one each to the left and right of the midline, were captured at 100 times magnification on a Zeiss Axioplan 2 Imaging system. Using spinal cord sections photographed at 10 times magnification, the cross-sectional area of the dorsal column was determined with ImageJ software. Total area of the 100 times images, excluding the grey matter and artefacts, were also calculated using ImageJ. These 100 times sections were adjusted for contrast and sharpness and converted to binary images, using Image J. These binary images were then imported to Image Pro software and a Gauss filter was applied. Axon parameters were acquired, using the counts/size function of Image Pro software, setting ranges for area, radius ratio and roundness to 0.2–90, 1–10 and 0–4, respectively. Areas not containing axons (inflammatory lesions and cells) were de-selected manually. The axon number was divided by the total ROI area, to obtain estimates of axon density per mm^2^. This method was validated against manual counting, and the average difference between methods was 223±193 axons/section (Data S3 B).

### Immunohistochemistry

For immunohistochemistry studies, at EAE day 16, a separate cohort of mice was deeply anesthetized with sodium pentobarbital (100 mg/kg i.p) and intracardially perfused with 20 mls 0.1 M PBS followed by 20mls 4% paraformaldehyde. The lumbar expansion of the spinal cord was removed, and then drop fixed in 4% paraformaldehyde solution for 30 min, placed into 20% sucrose/PBS solution overnight, oriented into Tissue-Tek Cryomolds (Sakura Finetek USA, Inc., Torrance, CA) in Tissue-Tek O.C.T., and frozen over iso-pentane. Cryostat sections were cut at 12 µm and slide mounted (Superfrost Plus; Menzel-Glaser, Braunschweig, DE). Tissue was collected from n = 9 CNTF KO/LIF HET and n = 7 CNTF KO/LIF KO at EAE day 16, because at this time EAE disease grades are significantly different between the groups, but few mice have become moribund.

For fluorescence immunohistochemistry, lumbar spinal cord sections were incubated overnight with rabbit anti-ionized calcium binding adaptor molecule 1 (IBA-1, 1/1000, Wako, Osaka, JP), rabbit anti-CD3 antibody (1/200, Dakocytomation, Glostrup, DK), rabbit anti-glial fibrilary acidic protein (GFAP; 1/200, Dakocytomation), or rat anti-NIMP-R14 to neutrophils (1/100, Abcam, Cambridge, MA), followed by goat anti-rabbit- or goat anti-rat- tetramethylrhodamine-5(and 6)isothiocyanate (TRITC)-conjugated secondary antibody (1∶200, Jackson ImmunoResearch, West Grove, PA), for 2 hours at room temperature. Cell nuclei were labelled with Hoechst 33342 (1∶5000, Invitrogen, Calsbad, CA) in PBS for 10 min at room temperature and slides were mounted with Mowiol solution (Merck) and coverslipped.

Images of stained spinal cord cross-section were captured on a Zeiss Axioplan 2 Imaging system. Cell counts were performed on images taken from the lateral funiculus and the dorsal column of the lumbar spinal cord, at 40×magnification. The images were imported into ImageJ software and manual counts and area measures were performed. Counts were expressed as number of cells per mm^2^. An average of 3 sections was assessed per mouse at intervals of 50 µm. Areas of inflammatory cell infiltrates were quantified from Hoechst 33258 (1∶5000 in 0.1 M PBS, 10 min; Molecular Probes, Carlsbad, CA) stained lumbar spinal cord sections as previously described [28]. The lesion area was also measured on sections captured at 5×magnification, using Image J.

### Adoptive Transfer Experimental Autoimmune Encephalomyelitis

Active MOG35–55 EAE was induced in 8 to 10 week old male and female CNTF KO/LIF HET (n = 6) and CNTF KO/LIF KO (n = 5) donor mice as described above. On day 10 post-immunization, the mice were anesthetized with sodium pentobarbital (100 mg/kg i.p) and intracardially perfused with 0.1 M PBS. The draining lymph nodes and spleen were then removed and placed into ice cold complete DMEM (11995, Gibco). Lymph nodes and spleen were ground and washed on to a 100 uM nylon sieve, and then centrifuged at 1500 rpm for 5 min. Red blood cells were lysed by re-suspending the cell pellet in ammonium chloride potassium carbonate lysis buffer (0.15 M NH_4_Cl, 10 mM KHCO_3_, 2 mM Na_4_EDTA dH20), 1ml per donor mouse, and incubating on ice for 5 min. The reaction was stopped by adding 9 ml complete media and mixing. The debris was left to settle to the bottom of the tube for 2 min, and cell suspension was transferred into a new tube and centrifuged at 1500 rpm for 5 min. The cell pellet was washed a further two times in PBS (Invitrogen) containing 0.5% FBS (Invitrogen), and then re-suspended in complete DMEM containing 50 ug of MOG_35–55_ peptide and 20 ng of IL-2, to obtain 6×10^6^ cells per ml for plating. The cells were then left to incubate for 48 h at 37C in a 5% CO_2_ incubator. After this time, the non-adherent T-lymphocyte cells were collected, washed twice with PBS containing 0.5% FBS twice, and injected into wild type donor mice at a concentration of 2×10^6^ cells in 0.1 ml PBS, i.p. per mouse (5 recipient mice per group). On the same day, the mice were injected with 300 ng pertussis toxin i.p., on the side opposite to the site of T-lymphocyte cell injection. Assessments of disease severity were performed daily as described above.

### Statistics

All statistical tests were performed using Graph Pad PRISM (v. 4.0; GraphPad Software Inc., La Jolla, CA). EAE grades were analysed using the Mann-Whitney rank sum test. Data from the pNF-H ELISA, cell counts and MRI were analysed using a 2-sample t-test or ANOVA followed by Student-Newman-Keuls post test. The experimenter was blinded to genotype for each experiment. P-values less than 0.05 were considered significant.

## Results

### The Presence of Endogenous CNTF and LIF is Associated with Improved Neurological Outcome during Acute EAE

**Figure 2:**
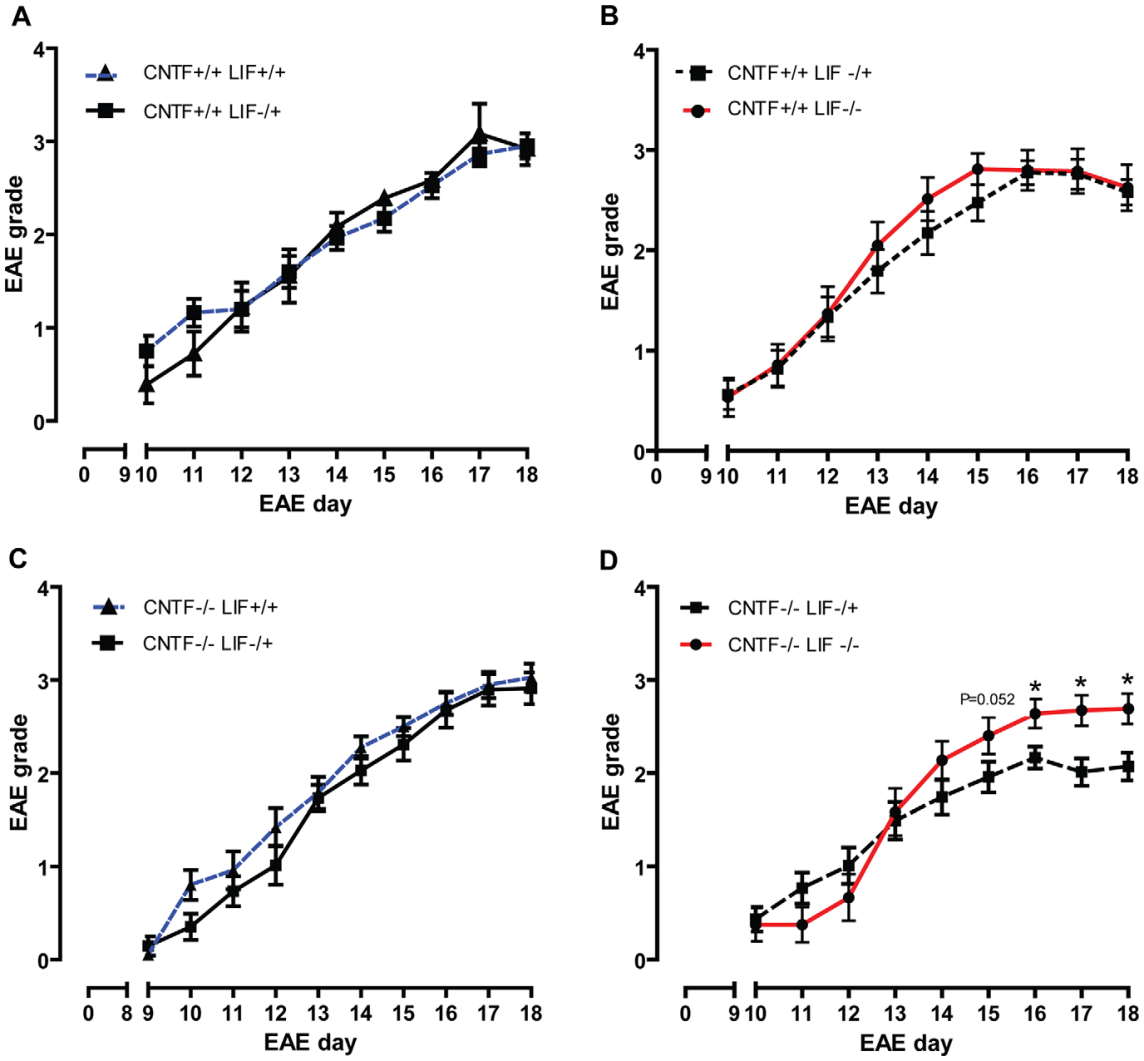
EAE disease severity grades. EAE disease severity scores for CNTF WT/LIF WT (n = 20) vs CNTF WT/LIF HET (n = 9) mice (A); CNTF WT/LIF HET (n = 30) vs CNTF WT/LIF KO (n = 21) mice (B); CNTF KO/LIF WT (n = 19) vs CNTF KO/LIF HET mice (n = 17) (C); and CNTF KO/LIF HET (n = 30) vs CNTF KOLIF KO (18) mice (D), over time. In CNTF gene deficient mice, the presence of the LIF gene was associated with reduced EAE disease severity scores relative to LIF gene deficient mice, from EAE day 15–18 (D)(*P<0.05). All data are presented as mean ± SEM.

Initially, we investigated the effect of endogenous CNTF and LIF on neurological outcome in EAE. As we only wanted to compare littermates to reduce cohort to cohort variability, we assessed EAE disease severity in a total of 4 different pairs of genotypes, as detailed below. Disease severity scores showed that, in the presence of CNTF, the genetic deletion of LIF does not influence acute EAE disease severity. This was observed for disease comparisons between CNTF WT/LIF WT vs CNTF WT/LIF HET mice (Fig. 2A) and CNTF WT/LIF HET vs CNTF WT/LIF KO mice (Fig. 2B). In the absence of CNTF, however, LIF KO mice showed significantly higher EAE disease severity scores compared with CNTF KO/LIF HET mice, from days 15–18 post induction (Fig. 2D). Importantly, at EAE day 18 the median grade for CNTF KO/LIF HET mice was 2.25 (range 1.5, 4) vs 2.75 (range 1.5, 4) for CNTF KO/LIF KO mice. Although this difference of two grading increments appears small, it should be noted that these grades represent mice with tail paralysis plus slightly abnormal gait vs mice with tail paralysis plus bilateral severe hindlimb paresis. Hence, this difference corresponds to a marked clinical difference between the groups. Relative to CNTF KO/LIF HET mice, the CNTF KO/LIF WT mice did not show a difference in their peak clinical disease severity (Fig. 2C), suggesting that one copy of the LIF gene is sufficient to confer maximal disease reduction in this context.

### Endogenous LIF is Associated with Improved Apparent Diffusion Coefficients and Anisotropy Ratios in the Optic Nerve Following EAE

**Figure 3:**
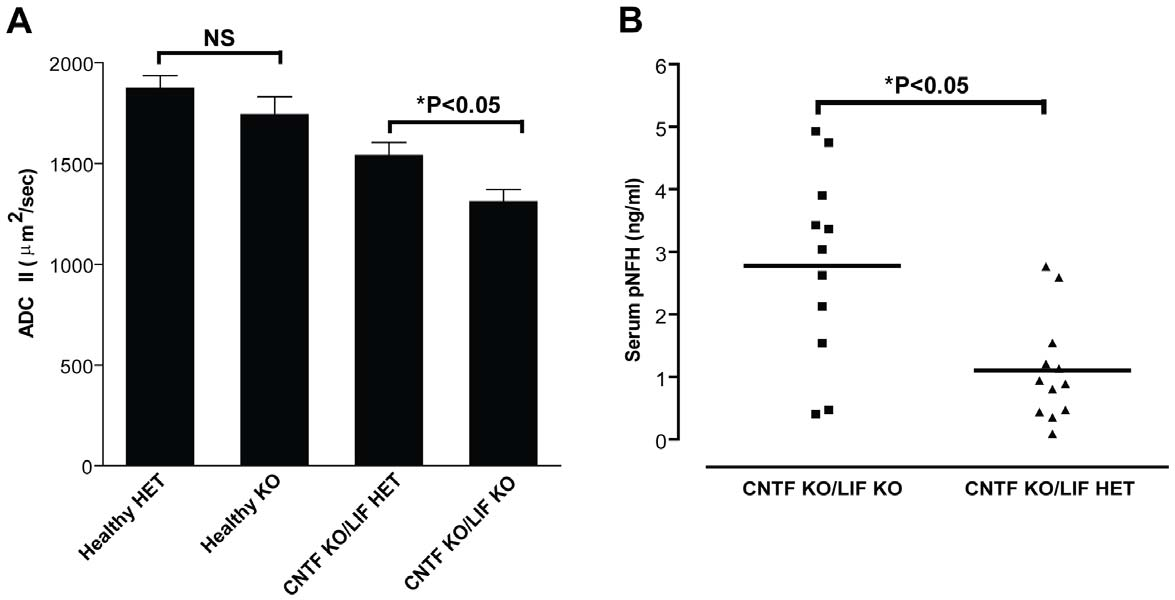
Optic nerve diffusion weight imaging outcomes. Acquired diffusion coefficient (parallel) (ADC II) values for optic nerves of Healthy (n = 8 per group) and EAE affected CNTF KO/LIF HET (HET; n = 10) and CNTF KO/LIF KO (KO; n = 8) mice (A). No significant differences were observed between healthy CNTF KO/LIF KO (healthy KO) and healthy CNTF KO/LIF HET (healthy HET) mice. Compared to healthy mice, ADC parallel (II), a proposed measure of axonal cytoskeleton integrity, was reduced in EAE affected mice (A) (*P<0.05). Optic nerve ADC II values were also significantly reduced in KO mice relative to HET mice with EAE (A) (*P<0.05), suggesting that deficiencies in CNTF and LIF are associated with axonal damage in the optic nerve with EAE. The double KO mice also show increased serum pNFH levels relative to HET mice (B; *P<0.05). Data for individual mice are presented, and group means are indicated by solid black lines.

Utilising in vivo MRI scanning in anaesthetised mice, the diffusion weighted imaging parameters ADC parallel and ADC perpendicular (to the long axis of the optic nerve) were used to investigate the integrity of optic nerve axons. No significant differences were observed between healthy CNTF KO/LIF KO and healthy CNTF KO/LIF HET mice (Fig. 3A; data not shown for ADC perpendicular). In EAE, ADC parallel was significantly higher in the CNTF KO/LIF HET mice (1540±207 µm^2^/sec) as compared to CNTF KO/LIF KO mice (1310±175 µm^2^/sec; Fig. 3A), whereas ADC perpendicular was not different (data not shown) indicating that CNTF KO/LIF KO mice exhibited diffusion MRI evidence of increased axonal damage, as previously validated [31].

### Endogenous LIF is Associated with Reduced Serum Levels of Neurofilament-H Following EAE

To ascertain whether the reduced disease severity that was observed in CNTF KO/LIF HET mice relative to CNTF KO/LIF KO mice was associated with evidence of reduced axonal injury, serum levels of the axonal cytoskeletal protein pNF-H were measured at the experimental endpoint (EAE days 18–21). Serum pNF-H levels were reduced approximately 2.5 fold in CNTF KO/LIF HET mice relative to CNTF KO/LIF KO mice (Fig. 3B, P<0.05), suggesting these mice had reduced axonal injury at peak disease.

### Endogenous LIF is Associated with Reduced Axonal Loss in the Optic Nerve and Spinal Cord during EAE

**Figure 4:**
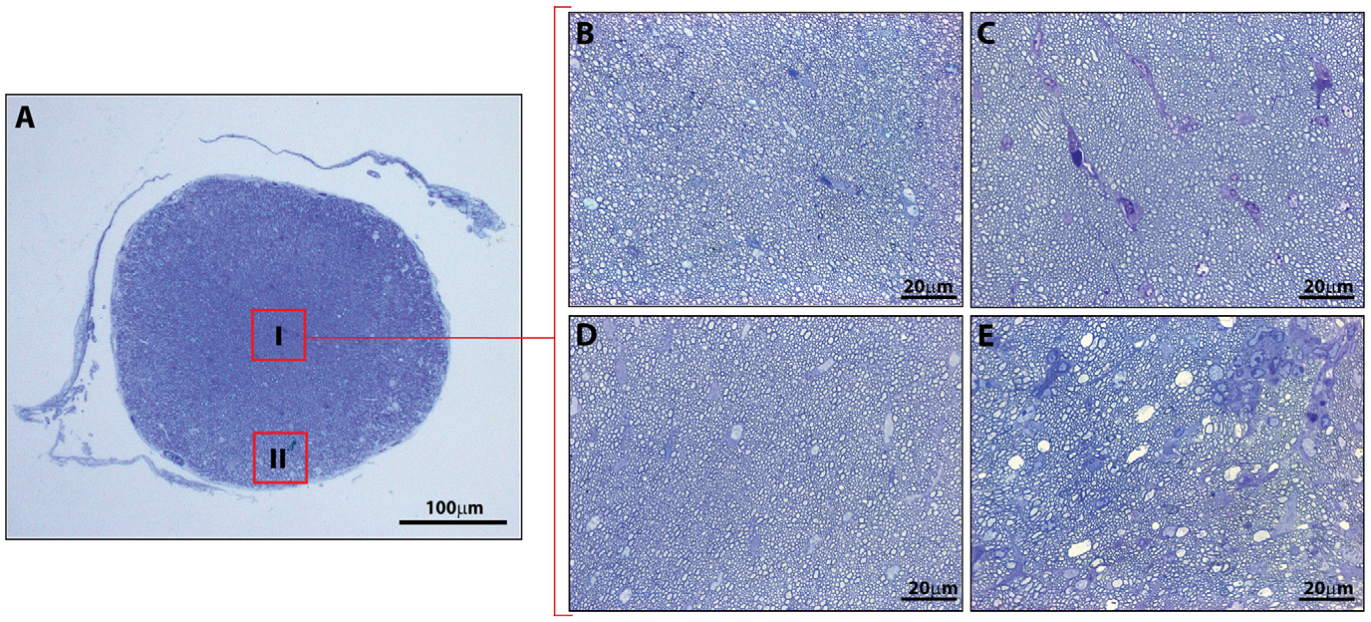
Representative images of methylene blue stained optic nerve sections used for analyses of axonal numbers. (A) Illustrates image selection of central (I) and peripheral (II) ROIs. Representative 100×magnification images taken in the centre of nerves from healthy CNTF KO/LIF HET (B), EAE induced CNTF KO/LIF HET (C), healthy CNTF KO/LIF KO (D), and EAE induced CNTF KO/LIF KO (E) mice. ROI’s were selected from within these larger images.

To confirm our MRI and blood assay results, we also used quantitative histology to assess axon densities in the optic nerve and spinal cord. On average, the total nerve cross-sectional area in healthy CNTF KO/LIF HET mice was significantly larger than the total nerve area of CNTF KO/LIF KO mice (Table 1). No other histological parameters were significantly different between healthy mice from each group. Following EAE, the average number of axons was 12.5% higher in CNTF KO/LIF HET mice compared to CNTF KO/LIF KO mice (2.0×10^3^±0.07/µm^2^ vs 1.75×10^3^±0.08/µm^2^, P<0.05, Table 1). Examples of optic nerve sections and ROIs used for these evaluations are given in Figure 4.

**Table 1:** Optic nerve histological parameters for CNTF KO/LIF KO vs. CNTF KO/LIF HET healthy and EAE induced mice.

Parameter (Average)	Healthy LIF HET	Healthy LIF KO	P value	EAE LIF HET	EAE LIF KO	P value
**Nerve area (mm^2^)**	0.07±0.002	0.06±0.003	0.02*	0.08±0.002	0.08±0.006	0.48
**Axon counts/ROI (×10^3^ µm^2^)**	1.92±0.09	2.14±0.01	0.16	2.00±0.07	1.75±0.08	0.02*

Comparison of key morphometric measures in the optic nerve between CNTF KO/LIF KO (LIF KO) and CNTF KO/LIF HET (LIF HET) mice in the presence and absence of EAE. Data are presented as mean ± SEM values for each parameter for each group. Region of interest (ROI) represents an area of 3480 µm^2^.

* P<0.05 by 2-sample t-test, assuming equal variances.

**Figure 5:**
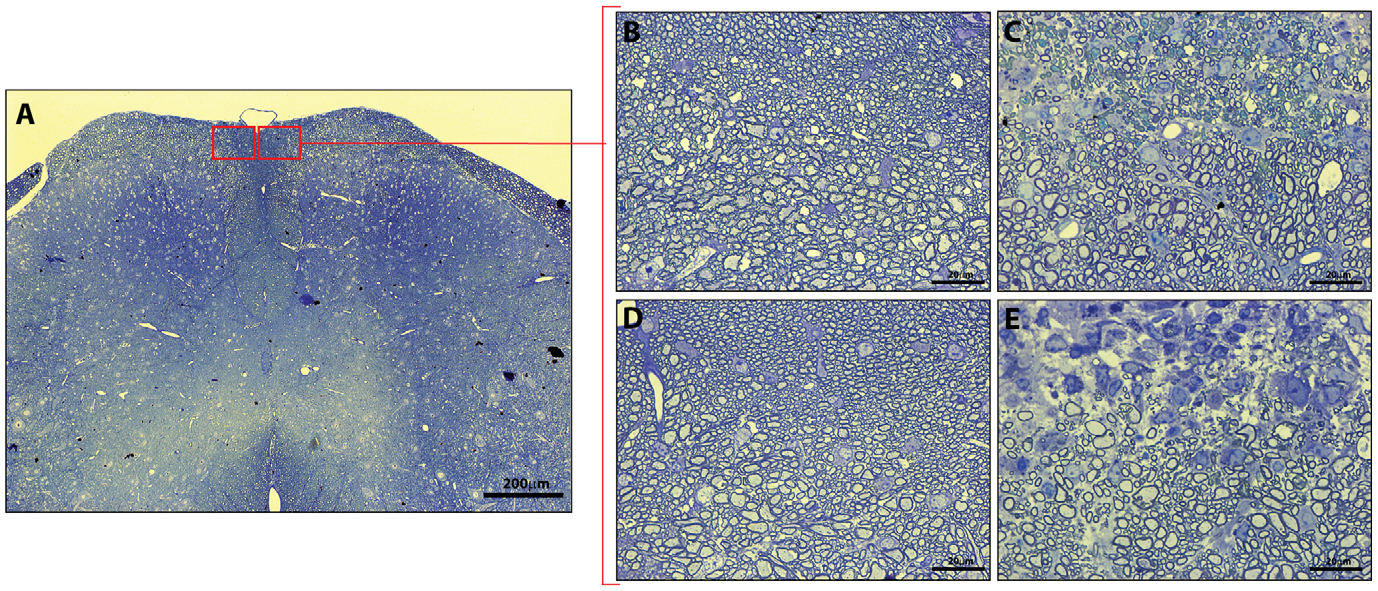
Representative images of methylene blue stained spinal cord sections used for analyses of axonal numbers. (A) Illustrates location of high magnification images captured within the dorsal column (red boxes). Representative 100×magnification images for CNTF KO/LIF HET (B), EAE induced CNTF KO/LIF HET (C), healthy CNTF KO/LIF KO (D), and EAE induced CNTF KO/LIF KO (E) mice.

There were no differences in spinal cord cross-sectional dorsal column size or axon density between healthy CNTF KO/LIF KO and CNTF KO/LIF HET mice (Table 2). Following EAE, axon densities were significantly higher within the dorsal column of CNTF KO/LIF HET mice compared to CNTF KO/LIF KO mice with EAE (1.19×10^5^ vs 1.02×10^5^±0.15×10^5/^mm^2^, P<0.05, Table 2). Representative examples of spinal cord sections and ROIs used for these evaluations are shown in Figure 5.

**Table 2:** Spinal cord histological parameters for CNTF KO/LIF KO vs. CNTF KO/LIF HET healthy and EAE induced mice.

Parameter (Average)	Healthy LIF HET	Healthy LIF KO	P value	EAE LIF HET	EAE LIF KO	P value
**Dorsal column area (mm^2^)**	0.16±0.01	0.18±0.02	0.44	0.16±0.01	0.18±0.01	0.18
**Axon counts/ROI (×10^5^ mm^2^)**	1.38±0.18	1.33±0.14	0.79	1.19±0.05	1.02±0.05	0.03*

Comparison of key morphometric measures in the dorsal column of the spinal cord, between CNTF KO/LIF KO (LIF KO) and CNTF KO/LIF HET (LIF HET) mice in the presence and absence of EAE. Data are presented as mean ± SEM values for each parameter for each group. Region of interest (ROI) represents the image area at 100×magnification excluding grey matter or artefacts.

* P<0.05 by 2-sample t-test, assuming equal variances.

### LIF does not Influence the Composition of Inflammatory Lesions or the Adaptive Transfer of EAE

**Figure 6:**
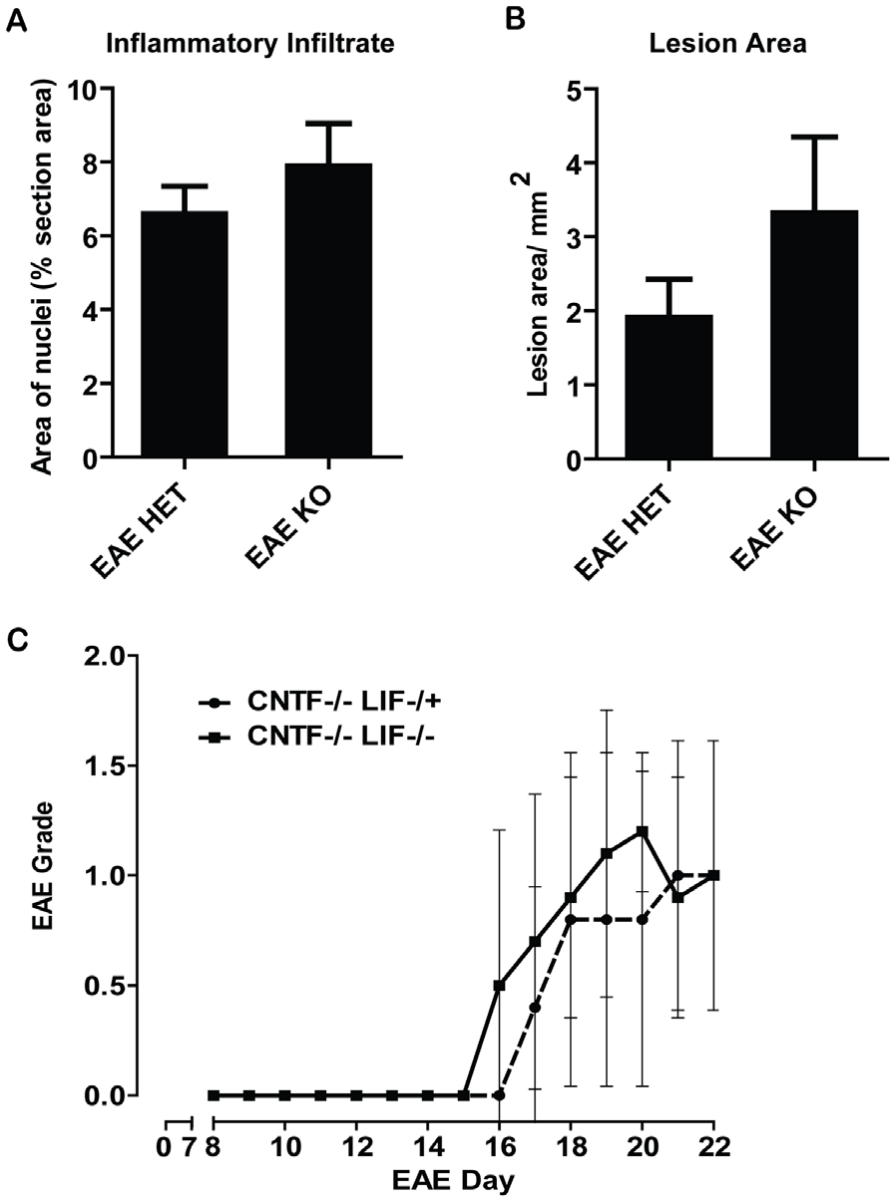
Analyses of inflammatory cell responses: Using images of Hoechst stained lumbar spinal cord sections captured at 20×magnification, the percentage of spinal cord area covered by cellular nuclei was measured using ImageJ, to indicate changes in cellularity resulting from inflammation (A). No differences were found between CNTF KO/LIF HET and CNTF KO/LIF KO mice during EAE day 15–16. Similarly, measures of the total area of inflammatory cell infiltrates in lumbar spinal cord sections captured at 5×magnification, showed no difference between the groups (B). Passive transfer EAE experiments showed no difference in EAE disease severity scores of CNTF WT/LIF WT recipient mice that received MOG primed T-lymphocytes harvested from CNTF KO/LIF HET or CNTF KO/LIF KO donor mice (C). Data are presented as mean ± SEM.

Histological analyses of lumbar spinal cord sections showed no difference in cellularity (Fig. 6A) or in the area of inflammatory cell infiltrates (Fig. 6B) for CNTF KO/LIF KO mice compared to CNTF KO/LIF HET mice following EAE.

**Figure 7:**
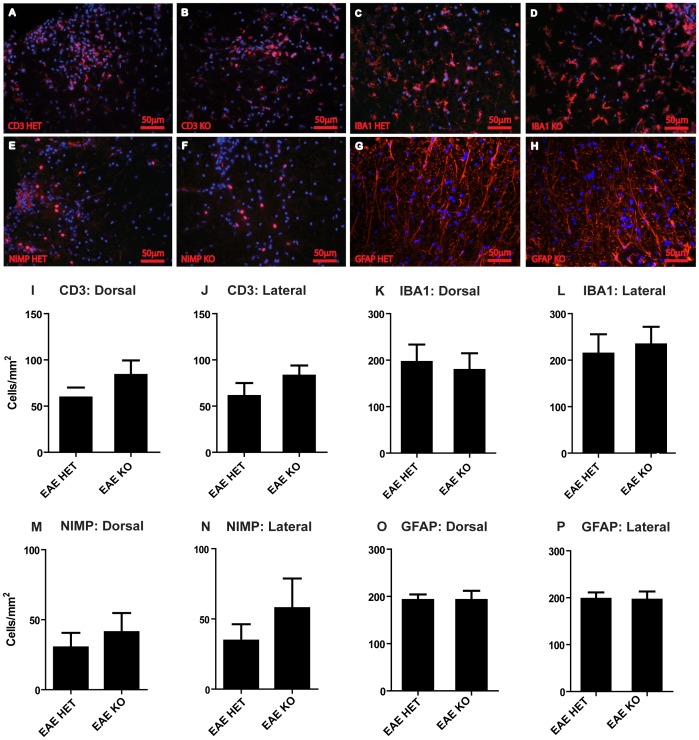
Analyses of CNS cell responses: Representative images of CD3 labelled T-lymphocyte cells (A, B), IBA1 labelled microglia/macrophages (C, D), NIMP-R14 labelled neutrophils (E, F) and GFAP labelled astrocytes (G, H), in lumbar spinal cord sections from CNTF KO/LIF HET and CNTF KO/LIF KO mice, respectively (all represented by red label, Hoechst stained nuclei in blue), at EAE day 16. Cell counts were conducted in the dorsal column (Dorsal) and at the lateral edges (Lateral) for CD3 labelled T-lymphocytes (I, J), IBA1 labelled microglia/macrophages (K, L), NIMP-R14 labelled neutrophils (M, N), and GFAP labelled astrocytes (O, P). No differences in cell numbers were found between the groups, Data are presented as mean ± SEM. Scale bar represents 50 µm.

Counts of CD3 expressing T-lymphocyte cells (Fig. 7A,B and I,J), IBA-1 expressing macrophages/microglia (Fig. 7C,D and K,L), NIMP-R14 reactive neutrophils (Fig. 7E,F and M,N), and GFAP expressing astrocytes (Figure 7G,H and O,P) also showed no difference between CNTFKO/LIF HET and CNTF KO/LIFKO mice following EAE. These results indicate that the presence of endogenous LIF does not influence the recruitment of inflammatory cells into the CNS during acute EAE lesion formation, or the cellular composition or size of the resultant inflammatory lesions.

To evaluate whether differences in disease severity between CNTF KO/LIF HET and CNTFKO/LIF KO mice could be attributable to differences in peripheral T-lymphocyte priming, adoptive EAE experiments were performed. No differences in disease induction or severity could be detected in CNTF WT/LIF WT recipient mice that received MOG primed T-lymphocyte cells from CNTF KO/LIF KO mice compared to CNTF KO/LIF HET mice (Fig. 6C). These results suggest that LIF and CNTF do not influence T-cell priming sufficiently to modulate EAE disease activity, consistent with the lack of difference in CD3 counts, lesion area, inflammatory cell density and lesional inflammatory cell composition between genotypes.

### The Deletion of gp130 from Oligodendrocyte Lineage Cells does not Worsen Acute EAE Disease Severity

**Figure 8:**
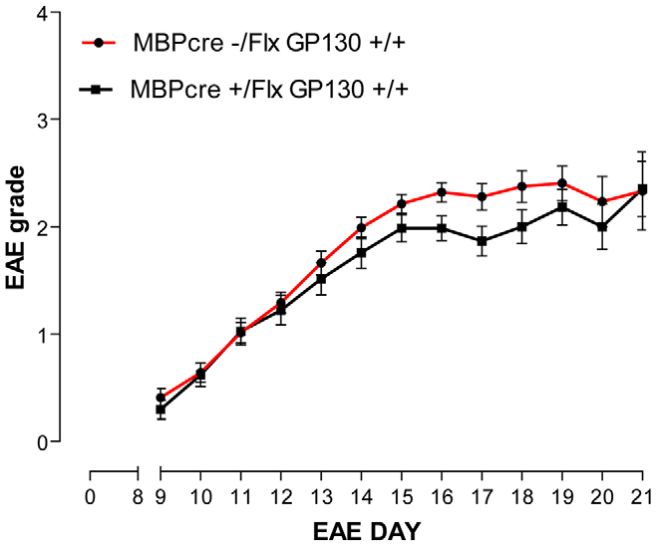
The effects of deleting gp130 from oligodendrocytes on EAE disease grade: Average EAE disease severity grades for MBP-cre gp130^fl/fl^ (black) vs gp130^fl/fl^ littermate control (grey) mice. The deletion of gp130 from oligodendrocyte lineage cells resulted in a transient improvement in EAE grade from EAE day 16–18 (P<0.05), but this effect was not sustained. Data are presented as mean ± SEM, and are analysed by Mann Whitney rank sum test.

To assess the role of gp130/LIFR signalling in the oligodendrocyte lineage on the severity of EAE, we generated conditional gp130 knockouts by crossing the gp130 floxed mouse line with mice expressing Cre recombinase behind the MBP promoter. Recombination efficiency was estimated at ∼90% by purifying the oligodendrocytes and assessing transcript levels by qPCR (see Data S2). The MBP-cre +ve gp130^fl/fl^ mice showed no significant change in EAE disease severity scores relative to MBP-cre -ve gp130^fl/fl^ littermate controls at peak disease (Fig. 8; EAE day 19–21). As there were no clinically significant differences in EAE severity between these cohorts, we did not proceed to histological evaluation. Based on these observations it is apparent that gp130 signalling in oligodendrocytes is not likely to mediate the neuroprotective effects of CNTF/LIF ligands in acute MOG_35–55_ EAE.

## Discussion

In this study, we show that in CNTF knockout mice, the presence of one copy of the LIF gene is associated with improved neurological outcome and significant relative axon protection in acute EAE, as measured by histology, biochemical assays, and diffusion weighted imaging of the optic nerves. In contrast, there was no difference in peripheral T-cell capacity to passively transfer EAE; the density of CD3-positive cells crossing the blood-brain barrier; or lesion size, number or composition between genotypes. This strongly suggested that the endogenous LIF and CNTF protected axonal integrity via activity in neural cell types.

In agreement with previous studies by Linker et al [18], we found that deletion of the LIF gene is insufficient to change neurological outcomes during the acute stages of EAE. It is apparent, however, that LIF can influence neurological outcome in acute disease when the CNTF gene is also deleted, suggesting that LIF signalling is redundant in this context. A similar observation was made in studies by Sendtner et al [13], who showed that deletion of the LIF gene alone did not influence facial motor neuron survival in post-natal mice subjected to facial nerve axotomy. In the absence of CNTF however, the deletion of LIF was shown to greatly potentiate neuronal death. Further study is required to determine whether CNTF signalling can also be fully compensated for by LIF in acute EAE, although previously published data suggests that deletion of this cytokine alone does not lead to worsening of acute EAE [19].

Importantly, for the first time, we convincingly show that endogenous LIF and CNTF are axon protective in acute EAE, using several specific measures of axonal injury and integrity. We found that a lack of endogenous CNTF and LIF resulted in a decrease in ADC parallel in the optic nerve by diffusion weighted MRI imaging, previously validated as a measure of optic nerve axonal integrity in healthy and EAE WT mice [31]; increased serum pNF-H levels; and reduced axon densities in optic nerve and spinal cord. This axonal protection was seen in the absence of a change in the extent of inflammatory infiltration, although we cannot exclude an effect on inflammatory cell activity. Importantly, although it has previously been described that CNTF KO/LIF KO mice on a mixed genetic background display spontaneous motor impairment and neuronal loss at 4–8 weeks of age [13], we were not able to observe any phenotypic changes in our healthy CNTF KO/LIF KO mice maintained on a C57Bl/6 background at up to 20 weeks, or any differences in axonal sizes or numbers relative to CNTF KO/LIF HET littermates at the same age that the EAE experiments were completed. Hence, it is highly unlikely that the differences that were observed in the present study are secondary to non-EAE mediated axonal loss [32].

Collectively, these results provide overwhelming evidence that endogenous LIF and CNTF signalling reduce acute EAE severity by limiting axonal damage and loss. Of note however, as previous studies have demonstrated that the expression of the LIF transcript is likely to peak reasonably early in the disease course (EAE day 14) and begins to decline by peak disease (EAE day 25) [24], it will be necessary to establish whether these axon-protective effects are sustained in more chronic disease where the expression of LIF could be reduced relative to acute disease. Furthermore, given that prior work shows therapeutic efficacy of LIF in the same mouse model [22], it is likely that this endogenous axon protective effect is sub-maximal, and hence, exogenous LIF could be used to augment this response.

One key potential effector cell for LIF/CNTF in EAE is the oligodendrocyte. These cytokines are powerful oligodendrocyte survival factors in vitro, and LIF therapy promotes oligodendrocyte survival in SJL/J mice with PLP139-151 induced EAE and also in murine spinal cord injury [22], [33]. However, we were not able to demonstrate any effect of conditional gp130 signalling deletion in oligodendrocytes on acute EAE disease severity. As EAE grades and spinal cord axonal injury are closely correlated in this model [28], we believe that in the absence of an observed clinical difference between mice with gp130 deletion in oligodendrocytes and control mice, an oligodendrocyte-mediated effect is very unlikely. Although surprising, these findings fit with our previous observations that oligodendrocyte loss and selective demyelination are minor pathological features of acute C57Bl/6 MOG_35–55_ EAE [28], [31]. It should however, be noted that detailed histological evaluations of axonal pathology were not conducted in these mice. Hence, it remains possible that gp130 signalling in oligodendrocytes promotes a minor protective effect on axons, insufficient to overtly influence disability outcome. Furthermore, our results do not examine potential recovery of axonal function beyond the acute phase of EAE.

Importantly, these experiments extend on recent studies by Haroon *et al* [24], who showed that synapsin I-cre mediated deletion of gp130 from CNS neurons did not influence the acute EAE disease course relative to that of floxed gp130 control mice; and that the deletion of gp130 from astrocytes, and potentially sub-populations of neurons, using GFAP-cre mediation resulted in more severe EAE in both acute and chronic stages of the disease. Hence, it appears increasingly likely that protective effects of the gp130 signalling cytokines in EAE are specifically mediated by astrocytic signalling. That said, the role of microglia/macrophage gp130 signalling in EAE has not been extensively investigated, and hence, they cannot be excluded as effector cells for CNTF/LIF in this disease. Interestingly, previous studies by Hendricks and co-workers [20] have demonstrated that LIF treatment can inhibit the production of pro-inflammatory mediators by mouse peritoneal macrophages in culture. Further, both LIF and CNTF have been shown to promote phagocytic activity in rat microglia [20], [34]. Although these effects have not been demonstrated *in vivo*, it is clear that the cell specific targeting of gp130 in macrophages/microglia is required to complete our understanding of the endogenous cellular targets contributing to the beneficial effects of LIF in EAE. More recently, it has also been demonstrated that recombinant LIF therapy can reduce the number of Th17 cells in the spleen and CNS of mice with EAE, and inhibit the differentiation of Th17 cells from human MS cases [35] in vitro. However, peripheral LIF antagonism using a recombinant soluble LIF receptor does not influence EAE severity [36], and LIF treatment of T-cells harvested from EAE induced animals to passively transfer EAE, did not alter pro-inflammatory cytokine profile or disease incidence or severity [22]. Hence, it seems unlikely that gp130 signalling in Th17 cells influences acute EAE severity.

Importantly, our study has provided evidence that endogenous LIF and CNTF, known to be produced in the EAE-affected spinal cord, significantly reduce inflammatory axonal loss and improve neurological outcome. This study confirms that the CNTF/LIF signalling pathway is an attractive neuroprotective therapeutic target for MS; and that LIF therapy could be useful as an adjunct to conventional immuno-modulatory or immune-cell trafficking inhibitory therapies. Finally, we present evidence against oligodendrocytes as direct effector cells of gp130 signalling in EAE, expanding on recent work that has also provided similar negative evidence for neurons and axons.

## Supporting Information

Data S1 **Methods for validating gp130 deletion from oligodendrocytes.** Protocol for primary mouse cortical oligodendrocyte cultures, and quantitative PCR validation of gp130 deletion from MBPcre^+^ gp130^fl/fl^ mice compared to MBPcre^−^ gp130^fl/fl^ control mice.
(DOCX)

Data S2 **Validating gp130 deletion from oligodendrocytes.** The mRNA levels of the floxed gp130 and gp130 transcripts in primary oligodendrocytes cultured from MBPcre^+^ gp130^fl/fl^ mice, were estimated to be reduced by over 90% relative to MBPcre^−^ gp130^fl/fl^ control mice (n = 6 mice cre positive mice pooled, and n = 4 cre negative mice pooled).
(TIF)

Data S3 **Validation of automated axon counting method.** Comparisons of automated (white bars) and manual axonal counting methods (black bars) revealed an average difference of 152.8±95.1 axons/section in the optic nerve (A), and 223±193 axons/section in the dorsal column of the spinal cord (B).
(TIF)
