# Supplementary material for: Leukemia Inhibitory Factor Protects Axons in Experimental Autoimmune Encephalomyelitis via an Oligodendrocyte-Independent Mechanism
Source: PLoS One. 2012 Oct 15;7(10):e47379. doi: 10.1371/journal.pone.0047379 (PMC3471848; doi:10.1371/journal.pone.0047379)
Supplement: Data S1 — Methods for validating gp130 deletion from oligodendrocytes. Protocol for primary mouse cortical oligodendrocyte cultures, and quantitative PCR validation of gp130 deletion from MBPcre+ gp130fl/fl mice compared to MBPcre− gp130fl/fl control mice. (DOCX) [file pone.0047379.s001.docx]

**S1**

**Validation of gp130 deletion using primary oligodendrocyte cultures**

For validation of gp130 deletion in MBPcre+ x gp130^fl/fl^ mice, oligodendrocyte lineage cells were isolated from P6 mouse forebrains using a panning purification method previously described by Cahoy *et al* ([2008](#_ENREF_7)). The cells were cultured according to methods established by Emery *et al* ([2009](#_ENREF_14)). To expand the cell population, cells were passaged after 7 days and plated on to poly-d-lysine coated T75 flasks. Once the cells reached confluence (approximately 3 days later), PDGF was withdrawn from the culture media for 24 hours to allow the cells to mature.

The floxed gp130 gene transcript expression was measured in cell lysates by quantitative polymerase chain reaction (qPCR). Primer sequences for qPCR were: 18S Forward 5’-CGGCTACCACATCCAAGGAA-3’, Reverse 5’-GCTGGAATTACCGCGGCT-3’; Floxed gp130 Forward 5’-TGGGCCGGAATTCACTTTT, Reverse 5’-CACGACTATGGCTTCTATTTCTCCTT-3’; gp130: Forward 5’- GCTGTAGAGCCTTCCTGAAACAG-3’, Reverse 5’- GATCACATTTCTCAGCCCTATGG-3’. The real time detection cycle was normalized to 18S in each case.
