# Supplementary figures and images for: Leukemia Inhibitory Factor Protects Axons in Experimental Autoimmune Encephalomyelitis via an Oligodendrocyte-Independent Mechanism
Source: PLoS One. 2012 Oct 15;7(10):e47379. doi: 10.1371/journal.pone.0047379 (PMC3471848; doi:10.1371/journal.pone.0047379)

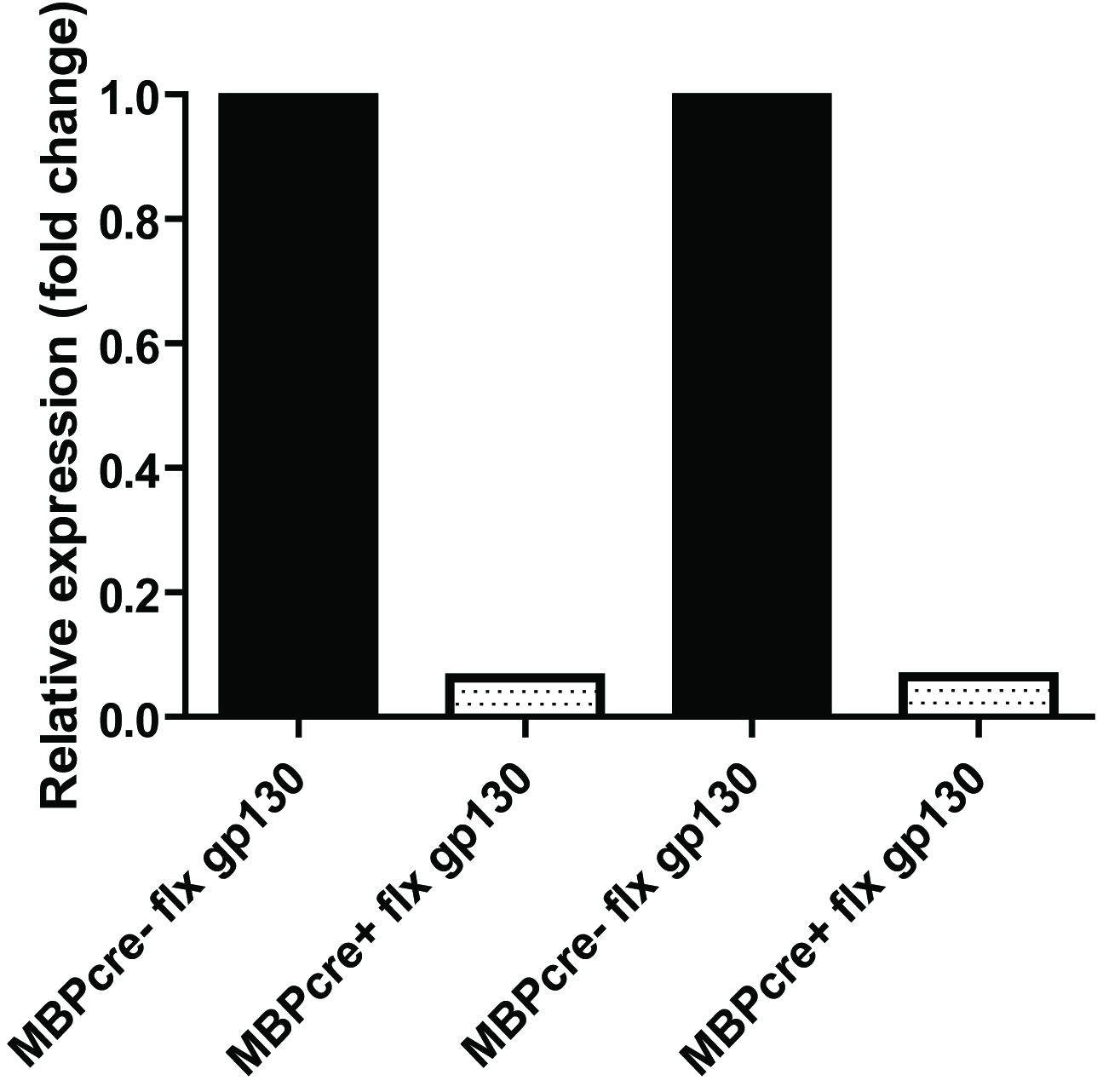

Supplement: Data S2 — Validating gp130 deletion from oligodendrocytes. The mRNA levels of the floxed gp130 and gp130 transcripts in primary oligodendrocytes cultured from MBPcre+ gp130fl/fl mice, were estimated to be reduced by over 90% relative to MBPcre− gp130fl/fl control mice (n = 6 mice cre positive mice pooled, and n = 4 cre negative mice pooled). (TIF) [file pone.0047379.s002.tif]

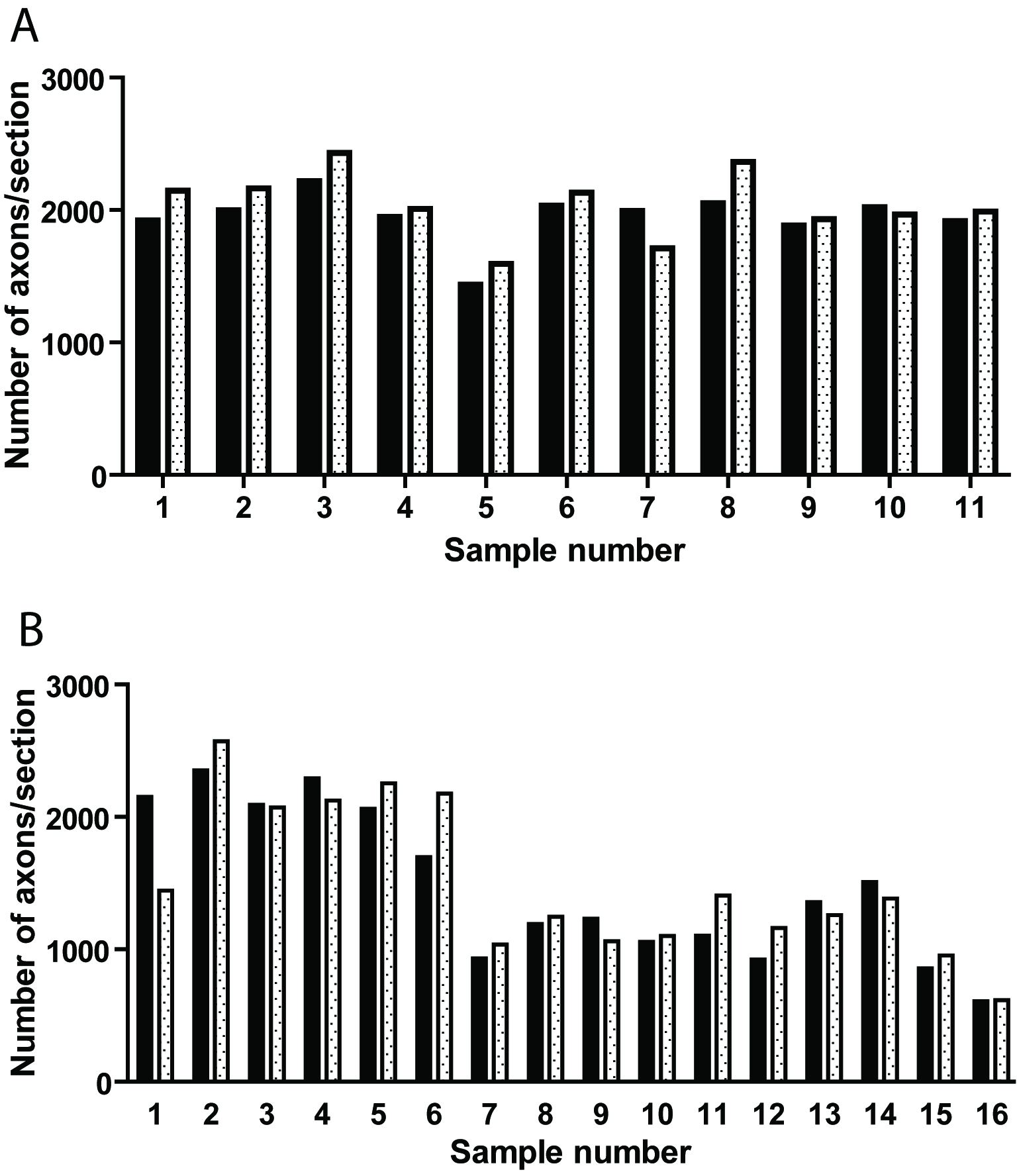

Supplement: Data S3 — Validation of automated axon counting method. Comparisons of automated (white bars) and manual axonal counting methods (black bars) revealed an average difference of 152.8±95.1 axons/section in the optic nerve (A), and 223±193 axons/section in the dorsal column of the spinal cord (B). (TIF) [file pone.0047379.s003.tif]
